# Toxin and capsule production by *Bacillus cereus* biovar *anthracis* influence pathogenicity in macrophages and animal models

**DOI:** 10.1371/journal.pntd.0012779

**Published:** 2024-12-23

**Authors:** Treenate Jiranantasak, Andrew P. Bluhm, Donald J. Chabot, Arthur Friedlander, Richard Bowen, Ian A. McMillan, Ted L. Hadfield, Airn Hartwig, Jason K. Blackburn, Michael H. Norris

**Affiliations:** 1 Spatial Epidemiology & Ecology Research Laboratory, Department of Geography, University of Florida, Gainesville, Florida, United States of America; 2 Emerging Pathogens Institute, University of Florida, Gainesville, Florida, United States of America; 3 United States Army Medical Research Institute of Infectious Disease, Fort Detrick, Maryland, United States of America; 4 Department of Biomedical Sciences, Colorado State University, Fort Collins, Colorado, United States of America; 5 Pathogen Analysis and Translational Health Group, School of Life Sciences, University of Hawaiʻi at Mānoa, Honolulu, Hawaiʻi, United States of America; Institute of Continuing Medical Education of Ioannina, GREECE

## Abstract

*Bacillus cereus* biovar *anthracis* (Bcbva) causes anthrax-like disease in animals, particularly in the non-human primates and great apes of West and Central Africa. Genomic analyses revealed Bcbva as a member of the *B*. *cereus* species that carries two plasmids, pBCXO1 and pBCXO2, which have high sequence homology to the *B*. *anthracis* toxin and polyglutamate capsule encoding plasmids pXO1 and pXO2, respectively. To date, only a few studies have investigated the effect of variations in Bcbva sporulation, toxin, and capsule synthesis on animal and macrophage pathogenicity compared to *B*. *anthracis*, therefore more research is needed to gain a better understanding of the pathogenesis of this emerging infection. Here, we report that Bcbva can multiply and vegetatively survive on nutrient-rich media for a minimum of six days while generating spores. Sporulation of Bcbva occurred faster and more extensively than *B*. *anthracis* Ames. Bcbva tended to secrete less protective antigen (PA) than *B*. *anthracis* Ames when cultured in growth medium. We found Bcbva produced a substantially higher amount of attached poly-ƴ-D-glutamic acid (PDGA) capsule than *B*. *anthracis* Ames when grown in medium supplemented with human serum and CO_2_. In a phagocytosis assay, Bcbva spores showed reduced internalization by mouse macrophages compared to *B*. *anthracis* Ames. Our research demonstrated that Bcbva is more virulent than *B*. *anthracis* Ames using two *in vivo* models, *Galleria mellonella* larvae and guinea pigs. Following that, the efficacy of the veterinary vaccine Sterne strain 34F2 against anthrax-like disease was assessed in guinea pigs. Sterne vaccinated guinea pigs had significantly increased anti-PA titers compared to the unvaccinated control group. Toxin neutralizing antibody titers in vaccinated guinea pigs correlated with anti-PA titers. This indicates the Sterne vaccine provides adequate protection against Bcbva infection in laboratory animals.

## Author summary

*Bacillus cereus* biovar *anthracis* (Bcbva) is a Gram-positive, spore-forming bacteria that causes anthrax-like disease in several animal species including non-human primates. It is listed as a select agent by the US Centers for Disease Control and Prevention (CDC). In sub-Saharan African rainforests, Bcbva is considered highly virulent with high mortality rates across multiple animal species. Bcbva shares key features with *B*. *anthracis*, the classical cause of anthrax, including spore production and environmental transmission. Our study demonstrates that Bcbva can replicate, sporulate, and persist in growth media for several days. We showed that Bcbva produced a greater amount of attached capsule when grown in culture media supplemented with human serum and CO_2_. Additionally, Bcbva caused higher mortality in laboratory animals infected with an equal number of spores compared to *B*. *anthracis* Ames. Nevertheless, Bcbva infection in animal models was prevented with the global standard Sterne anthrax vaccine. Further study should focus on the Bcbva lifecycle and transmission pathways in nature to elucidate new preventative strategies for controlling disease outbreaks.

## Introduction

Anthrax occurs nearly worldwide in livestock and wildlife with spillover into humans [1–3]. The disease mainly affects wild and domestic herbivores and is characterized by septicemia and sudden death [4]. Ungulates are frequently infected and are thought to acquire infection by ingestion of contaminated anthrax spores from the environment during feeding [4]. The primary routes of anthrax infection are the cutaneous, inhalational or gastrointestinal routes. Animals die from septicemia due to high concentrations of bacteria and robust secretion of anthrax toxins [5]. The carcasses release vegetative cells of *B*. *anthracis* in bloody discharge and body fluids emanating from the nostrils, mouth and anus into the environment after the host succumbs [6]. During decomposition, the vegetative cells within the infected carcasses may be exposed to the environment and spore forming conditions by scavenger animals or gas build up that rupture the abdomen [4,6]. Spores contaminate the area and can persist in the soil for years, creating a locally infectious zone [7,8]. Our previous study found that *B*. *anthracis* can multiply outside of the soil and persist on environmental substrates including rocks and leaves for at least seven days in controlled experiments [9].

For classical anthrax, the most effective intervention is sustained, preemptive veterinary vaccine programs for high-risk animal populations in enzootic areas [10]. In sub-Saharan Africa where anthrax risk is high, most livestock vaccination rates are extremely low [1]. Many anthrax vaccination programs are typically implemented reactively after an outbreak starts [1,11]. Currently, Sterne strain 34F2 is the most commonly used vaccine strain for livestock [12]. It is an attenuated vaccine based on a *B*. *anthracis* strain lacking the pXO2 plasmid but maintaining pXO1, an acapsular toxigenic live spore vaccine [13]. Once vaccinated, animals usually develop humoral immunity within 4 weeks [14]. Outside of livestock around the world, off label Sterne vaccine is used in some wildlife [15,16]. Annual vaccination is recommended as the immunity is not long-lived, yet over the last century, successful vaccination campaigns helped reduce the incidence of anthrax in livestock [12,17,18] and in parallel, human disease [19].

Until recently, non-human primate deaths from anthrax were rarely reported [20–22]. Increased anthrax rates in non-human primates have coincided with the discovery of *Bacillus cereus* biovar *anthracis* (Bcbva). Bcbva is the cause of anthrax-like disease in a variety of animal species including chimpanzees (*Pan Troglodytes*) [21,22], gorillas (*Gorilla gorilla*) [22], king colobus monkeys (*Colobus polykomos*) [23], sooty mangabeys (*Cercocebus atys*) [23], duikers (*cephalophus spp*.) [24], mongooses (*Herpetes spp*.) [24], elephants (*Loxodonta African*) [25], goats (*Capra hircus*) [25] and porcupines (*Atherurus spp*.) [24] among other animals in West and Central Africa. Bcbva was first discovered as a cause of chimpanzee mortality in Taї National Park, Côte d’Ivoire in 2001 [21]. Later, there were additional great ape deaths from Bcbva infection in Dja Reserve, Cameroon in 2004 [22]. Subsequently, Bcbva was found to be more widely distributed through the tropical forests of sub-Saharan African as it was detected in a moribund domestic goat in Democratic Republic of the Congo, and in great apes and an elephant in Central African Republic [25]. A recent study revealed more than 38% of the carcasses found in Taï National Park from 2001 to 2015 were associated with Bcbva [24]. The detection of low antibody rates and high anthrax-like mortality in wildlife across multiple species suggested that Bcbva is highly virulent and the causative agent in sub-Saharan African rainforests [26]. While no human anthrax cases caused by Bcbva have been reported nearly 10% of sera from humans living in Taï National Park tested positive for antibodies against a 35-kDa Bcbva-specific secreted protein, pXO2-60 [5,27]. Most study participants had a history of animal contact, but it was not significantly correlated with Bcbva seroprevalence [27]. This differs from *B*. *anthracis* exposure, where human cases are associated with handling infected animals or carcasses [28,29]. Whether these differences are the consequence of virulence factor diversity, human exposure, or lack of diagnostic capacity in the outbreak areas remains to be elucidated.

*Bacillus cereus* biovar *anthracis* is a Gram-positive, spore forming, rod-shaped bacterium closely related to *B*. *cereus* and *B*. *anthracis*. It harbors a *B*. *cereus*-like chromosomal background but contains two virulence plasmids, pBCXO1 and pBCXO2. The plasmids are homologous to pXO1 and pXO2 of *B*. *anthracis* at 99–100% nucleotide identity [25,30]. Plasmid pBCXO1 encodes the three toxin components, lethal factor (LF), edema factor (EF), and protective antigen (PA), whereas pBCXO2 is responsible for production of the poly-ƴ-D-glutamic acid (PDGA) capsule, respectively [20,22,25,30,31]. Besides PDGA capsule, Bcbva produces a hyaluronic acid capsule similar to *B*. *cereus* encoded by *hasACB* on the pBCXO1 plasmid [30,31]. Both PDGA and hyaluronic acid capsule are co-regulated by the global transcriptional regulator, AtxA [31,32]. Conversely, *B*. *anthracis* cannot generate the hyaluronic acid capsule due to a frameshift mutation in *hasA* causing premature termination of translation [31,33,34] and PDGA capsule expression is only activated by AtxA in the presence of CO_2_ and bicarbonate [31,32]. During anthrax infections, *B*. *anthracis* vegetative cells secrete two exotoxins which are required for pathology and mortality. Lethal toxin (LT) is comprised of protective antigen (PA) and lethal factor (LF), while edema toxin (ET) consists of PA and edema factor (EF) [20,30]. Upon toxin secretion, PA binds to the receptor on the host cells and is cleaved into truncated PA monomers by a furin-like protease enabling pre-pore formation. PA heptamers competitively bind with LF and EF to form complexes allowing their translocation into the cell by endocytosis. Once inside of the host cell, the PA heptamers form a pore at the surface of the endosome resulting in release of LF and EF into the host-cell cytoplasm. LT causes host lethality through systemic action of the LF zinc metallopeptidase activity which cleaves mitogen-activated protein kinase-kinases and other peptide hormones leading to shock and death [35,36]. ET causes edema attributed to the activity of EF as a calmodulin-independent adenylate cyclase which raises intracellular cyclic AMP levels [37,38]. Like *B*. *anthracis*, previous studies demonstrated Bcbva from both Côte d’Ivoire and Cameroon expressed PA by growing bacteria in bicarbonate medium supplemented with CO_2_ [20,31]. Phenotypically, both Bcbva and *B*. *anthracis* strains are non-hemolytic in contrast to typical *B*. *cereus* [20]. Bcbva strains have variable motility characteristics and are resistant to ƴ-phage, while *B*. *anthracis* is non-motile and susceptible to ƴ-phage [20]. In addition, Bcbva exhibits a null-*plcR* genotype like *B*. *anthracis* while the *plcR* transcriptional regulator in *B*. *cereus* is functional, affecting the hemolysis phenotype [30]. *Bacillus anthracis* is usually sensitive to β-lactam antibiotics, such as penicillin, and quinolones including ciprofloxacin or doxycycline whereas most Bcbva show resistance to β-lactam antibiotics and intermediate sensitivity to amoxicillin-clavulanic acid [20,25,27,30]. Beyond these genetic and phenotypic characteristics, few studies have explored the sporulation properties and kinetics, pathogenic mechanisms in infected macrophages, and lethal doses of Bcbva in animal models, necessitating further investigation [31,39].

In the United States, *B*. *anthracis* is stringently controlled by the Centers for Disease Control and Prevention (CDC) and is considered an overlap select agent because of its potential threat to human and animal health [40]. High genetic similarity between the virulence plasmids of Bcbva and *B*. *anthracis*, and the ability of Bcbva to cause anthrax in animals have led to its inclusion on the US CDC Tier 1 select agent list [41]. Previous studies revealed dormant Bcbva spores persist in animal components such as bones and teeth for several decades [23,24] and, to date, there is no licensed vaccine available for Bcbva infection in animals.

Understanding the pathogenesis of Bcbva allows for development and testing of efficient intervention strategies to prevent anthrax-like disease. The objectives of this study were to compare sporulation, capsule production, and virulence of Bcbva from Côte d’Ivoire and *B*. *anthracis* Ames using *in vitro* and *in vivo* models. The median lethal dose (LD_50_) of Bcbva and mortality data for inhalation route in small animals were investigated. Additionally, since the capsule and toxins produced by Bcbva are homologous to those expressed by *B*. *anthracis*, we examined whether the Sterne vaccine could provide sufficient protection against anthrax-like disease cause by Bcbva in inhalation-infected animals, which may be an essential consideration for developing any sustained livestock or wildlife anthrax preventive program in Bcbva enzootic areas.

## Materials and methods

### Ethics statement

Animal challenges were performed at the Rocky Mountain Regional Biosafety Laboratory at Colorado State University under Colorado State University Institutional Animal Care and Use Committee (IACUC) protocol #1561 following ABSL3 practices and procedures.

### Bacterial strains

Strains Bcbva UFBc0009 (replicate UFBc0009.1) and *B*. *anthracis* Ames UF00738 (replicate UF00738.1) are curated in the Martin E. Hugh-Jones *Bacillus anthracis* Collection at the Emerging Pathogen Institute, University of Florida. Bcbva UFBc0009 was recovered from mandible tissue and associated teeth from a monkey carcass (*Cercocebus atys*) in Taï National Park, Côte d’Ivoire between 1993 to 1994 [23]. The original strain of *B*. *anthracis* Ames UF00738 was isolated from a heifer which died of anthrax in Jim Hogg County, Texas in 1981 [42,43] and has long been used as a lab reference strain. All bacterial strains were manipulated using biosafety level 3 (BSL3) standard operating procedures following the Biosafety in Microbiological and Biomedical Laboratories 6^th^ edition [44].

### Spore production and purification

Starter cultures were inoculated in 3 mL of brain heart infusion (BHI) broth (Difco, BD Life Sciences, Sparks, MD, USA) in sterile 15 mL tubes with 0.22 μm ventilated caps (CELLTREAT Scientific Products, Pepperell, MA, USA). The inoculations were shaken at 220 rpm at 37°C overnight. 200 μl aliquots of culture were spread on Difco sporulation media (DSM) agar [45] with a total of 10 plates for each strain and incubated at 37°C for 6 days. The sporulation efficiency of each plate was measured with wet mount microscopy and was always ≥99% complete prior to collection. Spores from each plate were harvested in 2 ml of ice-cold sterile MilliQ water and combined into a single tube. The spore suspension was pelleted by centrifugation at 4000 x *g* at 4°C for 10 min. The supernatant was removed, and the pellets were purified through a sodium diatrizoate gradient (MP Biomedicals, LLC., Slon, OH, USA) as previously described [46,47]. The spore pellets were resuspended in 95% ethanol and incubated at room temperature for 1 h with 1 min of vortexing at 15-min intervals to kill any residual vegetative bacilli. The pellets were washed 3 times and resuspended with ice-cold sterile MilliQ water. The spore suspensions were kept at 4°C until further use. The number of viable spores was identified by serial dilution in sterile 1x phosphate buffer saline with 0.05% (v/v) Tween-20 (PBST), plated on tryptic soy agar (TSA) (Research Products Inter, Mount Prospect, IL, USA), and incubated at 37°C overnight.

### Sporulation assay

Strains were inoculated and grown to stationary phase in BHI broth shaken at 220 rpm at 37°C overnight. The cultures were adjusted to an OD_600_ of 1 and subcultured at 1:100 in 3 ml of heart infusion broth (HIB) (Research Products International, Mt. Prospect, IL, USA) in duplicate. The inoculum was incubated at 37°C until processed at 1-, 2-, and 6-day post incubation. At each timepoint, 100 μl aliquots of each culture were serially diluted in PBST, 100 μl from each dilution was plated on TSA. Another 300 μl aliquots of each inoculation were collected and mixed with 700-μl of absolute ethanol to obtain a final concentration of 70% (v/v). The mixtures were incubated at room temperature and vortexed for 1 min at 15 min intervals for 1 hour to ensure homogenous exposure of ethanol throughout the mixtures. Ethanol mixed samples were serially diluted in sterile PBST, and 100 μl from each dilution was plated on a TSA. All plates were incubated at 37°C overnight to enumerate bacterial colony forming units (CFU). The total number of bacterial cells and spores was determined by multiplying CFU by the dilution factor. This assay was conducted in two independent experiments, and three replicate cultures of bacterial strains were grown in each experiment.

### Internalization assays

Murine macrophage cell lines RAW264.7 (American Type Culture Collection, ATCC) was seeded in a 96-well CellBIND plate (Corning, Corning, NY, USA) at 25,000 cells/well in Dulbecco’s Modified Eagle Medium (DMEM)-high glucose + L-glutamine (Gibco, Life Technologies, Grand Island, NY, USA) supplemented with 10% (v/v) fetal bovine serum (FBS) (R&D Systems, Flowery Branch, GA, USA). The plate was incubated at 37°C with 5% CO_2_ overnight to allow monolayer formation. RAW264.7 cells were infected in triplicate with Bcbva UFBc0009 and *B*. *anthracis* Ames UF00738 spores at a multiplicity of infection (MOI) of 20 in 50 μl of DMEM without FBS for 45 min to enhance gravity assisted contact between spores and macrophages. Spores do not germinate in DMEM media alone [48–50]. After the infection, DMEM containing 10% FBS, 0.5 mM L-alanine, 1 mM inosine, and 100 μg/ml gentamicin was added and incubated for 15 min. L-alanine and inosine are germinants and play an important role in initiating the germination of *B*. *cereus* and *B*. *anthracis* endospores [51,52]. In addition, the germination of *B*. *anthracis* spores is faster with the addition of serum to the cell culture growth medium [48]. The combination of L-alanine, inosine, and serum can cause *B*. *anthracis* and *B*. *cereus* spores to germinate within 15 min [48,51,53]. Upon spore germination, the extracellular vegetative cells were killed by gentamycin [50]. Then, the supernatant was removed, and macrophages were incubated for another 2 h in DMEM plus 10% FBS. The cells were washed with phosphate buffer saline (PBS) 3 times between each step. To lyse the cells, 0.1% (v/v) TritonX-100 (Amresco, Solon, OH, USA) was added for 15 min, and supernatants were serially diluted in PBST. All serial dilutions were plated on TSA for counting the number of bacterial cells.

### Lactate Dehydrogenase (LDH)-Mediated Cytotoxicity Test

During anthrax infection, the excessive toxin production during bacterial multiplication leads to cellular damage and cell death. Cytotoxicity is commonly determined by measuring the amount of LDH, a stable cytoplasmic enzyme, that rapidly released from damaged cells into the cell culture supernatant [54]. To determine LDH activity, RAW264.7 cells were seeded as described above. Spore suspensions of Bcbva UFBc0009 and *B*. *anthracis* Ames UF00738 in DMEM were added to macrophages at an MOI of 20 in six replicates for each strain and incubated for 45 min. After incubation, DMEM supplemented with 10% FBS, 0.5 mM L-alanine, 1 mM inosine, and 100 μg/ml gentamicin was added and incubated for 15 min. The plate was washed with PBS three times between each step. Fresh DMEM containing 10% FBS was added and incubated further for 16 h. For detecting a maximum LDH activity, 10 μl of 10X lysis solution was added into one set of six replicate wells. Next, 10 μl of sterile ultrapure water was added into another set of six replicate wells to measure spontaneous LDH activity. After incubation, 50 μl of supernatant from each well were transferred to a fresh 96-well plate for measurement of LDH by using CytoTox 96 Non-Radioactive Cytotoxicity Assay following the manufacturer ‘s recommendations (Promega Corporation, WI, USA). Briefly, 50 μl of CytoTox 96 reagent was added to each well and incubated for 30 min at room temperature. Then, 50 μl of stop solution was added to each well to stop the reaction. The level of LDH was detected by measuring the absorbance signal at 490 nm in a spectrophotometer (Agilent Biotek Synergy HTX Multi-Mode Microplate Reader, Agilent Technologies, Inc., Santa Clara, CA, USA).

### Protective antigen production

Bacterial cultures were grown in BHI broth at 37°C and shaken at 220 rpm overnight after which they were adjusted to an OD_600_ of 1 and subcultured at 1:100 in 3 ml of HIB broth or BHI broth in duplicates. The common components across these media are heart extract, digastric enzyme, and electrolytes. BHI is supplemented with glucose as a source of sugar. The comparison of cultures grown in HIB and BHI media was to observe how glucose levels could impact PA secretion without elevation of CO_2_/bicarbonate in Bcbva compared to *B*. *anthracis*. Briefly, the cultures were supplemented with cOmplete, EDTA-Free Protease Inhibitor Cocktail (Roche Diagnostics GmbH, Mannheim, Germany), to a final concentration of 1x following the manufacturer’s instruction. At 24 h post incubation, 1 ml of each culture was collected and spun down at 14,000 x *g* for 10 min. The supernatant was harvested and filtered through low volume 0.45 μm PVDF filters (Millipore Sigma) followed by Spin-X 0.22 μm filters at 10,000 x *g* for 1 min. The filtered supernatant was collected and stored at -80°C for further PA measurement. The level of PA was quantified using the Anthrax Protective Antigen 83 ELISA Kit according to the manufacturer’s recommendations (Alpha Diagnostic International, San Antonio, TX, USA). The absorbance was read at 450 nm and 630 nm using a microplate reader (Agilent Biotek Epoch 2, Agilent Technologies, Inc., Santa Clara, CA, USA).

### Capsule staining

Strains were streaked and grown on nutrient broth-yeast extract (NBY) agar plates supplemented with 0.7% (w/v) sodium bicarbonate (NaHCO3) (Fisher Scientific, Faire Lawn, NJ, USA). The plates were incubated in 20% CO_2_ at 37°C for 24 h. To visualize the bacterial capsule, India ink staining was performed by adding a drop of India ink (Becton, Dickson and Company, Sparks, MD, USA) and mixing with a loop of culture on a clean glass slide. The slide was covered with a cover slip and the edges sealed with nail polish. The bacterial capsule was examined under the microscope with oil immersion (100x) objectives (Evos XL Core Imaging System, Life Technologies Corporation, Bothell, WA, USA).

### Indirect ELISA for detecting attached and secreted PDGA capsules

The starter cultures were grown to mid-log phase in BHI broth at 37°C while shaken at 220 rpm. OD_600_ was adjusted to 1 and subcultured at 1:100 to inoculate 3 ml of DMEM with 10% (v/v) normal human serum (NHS) (MP Biomedical, LLC, Solon, OH, USA) in triplicate and supplemented with 20% CO_2_ to stimulate the formation of capsules. DMEM supplemented with serum and CO_2_ can mimic the nutritional milieu found within host’s tissues, providing insights into bacterial virulence under conditions like *in vivo* settings. The cultures were shaken at 220 rpm and incubated at 37°C for 24 or 48 h. At each time point, 1 ml of each culture was harvested and centrifuge at 5,000 x *g* for 10 min. The supernatants containing secreted capsule were collected. To harvest the attached capsules, the cell pellets were resuspended in sterile MilliQ water and heated at 100°C for 20 min to lyse cells and release the cell membrane attached capsule. The cell suspensions were centrifuged at 5000 x *g* for 10 min followed by collecting the supernatant harboring the released attached capsules. All samples were stored at -80°C. To assess the level of attached and secreted PDGA capsule from Bcbva UFBc0009 and *B*. *anthracis* Ames UF00738, supernatants at 24- and 48-h post inoculation were analyzed using ELISA. Briefly, Immulon 4HBX flat-bottomed 96-well microtiter plates (Immulon, Thermo Scientific, USA) were coated in duplicate with 100 μl per well of either a 1:50 dilution of supernatant containing secreted capsules or 1:200 dilution of supernatant carrying attached capsules in sterile 1x PBS. A known concentration of purified capsule [55] was two-fold serially diluted and used to coat wells of a 96-well microtiter plate with 100 μl per well in duplicate to create a standard curve. All plates were incubated overnight at 4°C and washed with 300 μl of PBST before proceeding. The antigen-coated plates were blocked with 300 μl of blocking solution (5% skim milk in PBST) for 1 h at room temperature. After blocking, 100 μl of 1:6000 diluted mouse anti-PDGA capsule IgM [56,57] in blocking solution were added to the wells and incubated for 1 h at room temperature. After incubation and washing, goat anti-mouse IgM conjugated with horse radish peroxidase (HRP) (Invitrogen, Camarillo, CA, USA) was diluted to 1:3000 in blocking solution, and 100 μl of diluted secondary antibody solution was added to each well and incubated for 1 h at room temperature. Finally, the antigen and antibody complexes were detected by adding 50 μl of 3,3’,5,5’ tetramethylbenzidine (TMB) (1-Step TMB ELISA Substrate Solutions, Thermo Scientific, IL, USA) and incubating for 30 min at room temperature. The reaction was stopped by adding 50 μl of 1 N hydrochloric acid (EMD Millipore Corporation, Burlington, MA, USA) and the absorbance was measured at 450 nm using microplate reader (Agilent Biotek Epoch 2, Agilent Technologies, Inc., Santa Clara, CA, USA). The standard curve from a known concentration of purified capsules was generated, and the concentrations of secreted and attached capsules in each sample were calculated.

### *Galleria mellonella* infection

*Galleria mellonella* larvae are commonly used as surrogate hosts for infectious disease models including *Burkholderia* spp., *Listeria* spp., *Pseudomonas* spp., *Staphylococcus* spp., *Mycobacterium* spp., *B*. *anthracis*, and pathogenic *B*. *cereus* strains [47,58–64]. Wax moth larvae have several benefits as they are easy to use, and the presence of an innate immune system mimics that of vertebrates [59,65,66]. To investigate the virulence of Bcbva UFBc0009 and *B*. *anthracis* Ames UF00738 in *G*. *mellonella*, larvae worms weighed between 100–250 mg were used in this study. Worms were purchased from Carolina Biological Supply Company (Burlington, North Carolina, USA) and used within 5 days of receipt. Bcbva UFBc0009 and *B*. *anthracis* Ames UF00738 spores were generated and purified as described above. Purified spores were diluted, plated, and enumerated before diluting to the desired inoculum of 10^7^ spores/ml in PBS. Inoculum were serially diluted to 10^3^ spores/ml and kept on ice until use. A total number of 120 worms were randomly assigned into six groups with 20 worms per group. A Hamilton 50 μl microsyringe with a 27-gauge needle was used for injections. Worms were briefly placed on ice to induce torpidity and reduce movement before injection. Each larva was injected with 10 μl of inoculum into the third distal left proleg as desribed previously [47]. Larvae were monitored at room temperature for 30 min before placing at 37°C. The survival of worms was checked at 24-, 48-, and 72 h post-infection by observing melanin pigment formation and no sign of movement when touched.

### Animal studies

Guinea pigs were chosen to determine Bcbva virulence because these animals have been widely used for investigation of anthrax pathogenesis and therapeutic efficacy [67–70]. Inhalation anthrax in guinea pigs shows similar infection mechanisms to those seen in non-human primates and humans [67,71]. Although death in guinea pigs often occurs within 2–4 days post infection, protection can be achieved through optimal vaccination [67,72]. Six-to eight-week-old male and female Dunkin-Hartley guinea pigs (Elm Hill Labs, Chelmsford, MA) were provided food and water ad libitum. In the LD_50_ studies, an equal number of eight-week-old female and male guinea pigs (n = 10 per group) were weighed then anesthetized with intraperitoneal ketamine-xylazine injections and challenged intranasally with spores in 0.5 ml of PBS. For the vaccine challenge experiments, six-week-old guinea pigs (n = 8 per group) were vaccinated subcutaneously with 1x10^6^ spores of the Sterne 34F2 veterinary vaccine (Colorado Serum Company, Denver, CO, USA). Four weeks later the animals were anesthetized, and their blood was collected for further analysis. The anti-PA titers from vaccinated and unvaccinated animals were quantified by using a guinea pig anti-anthrax protective antigen 83 (PA83) ELISA kit according to the manufacturer’s recommendations (Alpha Diagnostic International, San Antonio, TX, USA). Serum from unvaccinated animals served as a control. Animals were then intranasally challenged with 100x LD_50_ of *B*. *anthracis* Ames spores (LD_50_ = 3.61x10^5^ spores determined for this study) or 100x LD_50_ of Bcbva spores (LD_50_ = 3.49x10^4^ spores) and followed for survival for 14 days. Early intervention endpoints were determined for all studies, and guinea pigs were humanely euthanized when moribund or at the end of study. Lungs and spleens were collected and processed to determine bacterial loads in organs.

### Anthrax lethal toxin neutralization assay

RAW264.7 cells were seeded as mentioned above and cultured in DMEM supplemented with 10% FBS and 10 mM HEPES (Fisher Scientific, Fai Lawn, NJ, USA) overnight. The toxin neutralization assay was performed as previously described [73]. Pooled serum samples from vaccinated guinea pigs before challenging with Bcbva UFBc0009 and *B*. *anthracis* Ames UF00738 spores were prepared at 200-, 1,200-, and 12,000-fold dilutions in DMEM supplemented with 10% FBS and 10 mM HEPES. Pooled serum samples from unvaccinated animals served as a control. The diluted serum samples were then incubated with 50 ng/ml PA (List Labs, Campbell, CA, USA) and 160 ng/ml LF (List Labs, Campbell, CA, USA) for 30 min before adding to RAW264.7 cells. The mixtures of cells, serum, and toxin were incubated for 6 h. After incubation, the supernatant was collected and further used for detection of cytolytic activity by using CytoTox 96 Non-Radioactive Cytotoxicity Assay following the manufacturer ‘s recommendations (Promega Corporation, WI, USA).

### Statistical analysis

The sporulation efficiency of Bcbva UFBc0009 and *B*. *anthracis* Ames UF00738 spores are presented as means with standard error of the mean (SEM) and are displayed in bar graphs. The Shapiro-Wilk test was used to analyze the sample distribution. The unpaired t-test was performed to determine significance difference of sporulation, cytotoxicity, macrophage internalization, and attached or secreted PDGA capsules in DMEM supplemented with 10% (v/v) NHS in 20% CO_2_ between Bcbva UFBc0009 and *B*. *anthracis* Ames UF00738. The significant difference of PA production between Bcbva UFBc0009 and *B*. *anthracis* Ames UF00738 in HIB and BHI media was determined by using the Mann-Whitney U test. A Kruskal-Wallis test with Dunn’s multiple comparison test was used to identify significant differences of anti-PA titers among vaccinated and unvaccinated groups. The survival of *G*. *mellonella* larvae and guinea pigs was plotted as a survival curve. The LD_50_ values of *G*. *mellonella* larvae infected with either Bcbva UFBc0009 and *B*. *anthracis* Ames UF00738 was determined and compared using nonlinear regression analysis. The LD_50_ values of *B*. *anthracis* Ames UF00738 infected guinea pigs were calculated using the sigmoidal non-linear regression half max calculations in GraphPad Prism fitting the curve to the log transformed group dosing plotted against survival probability at that dose. The log-rank test was used to identify the significance between vaccinated and unvaccinated guinea pig groups. Graph generation and statistical analyses were done in GraphPad PRISM 10 software (GraphPad Software, San Diego, California, USA) with significant level at *ρ* < 0.05.

## Results

### Sporulation, cytotoxicity, and macrophage internalization of Bcbva and *B*. *anthracis* spores

**Fig 1:**
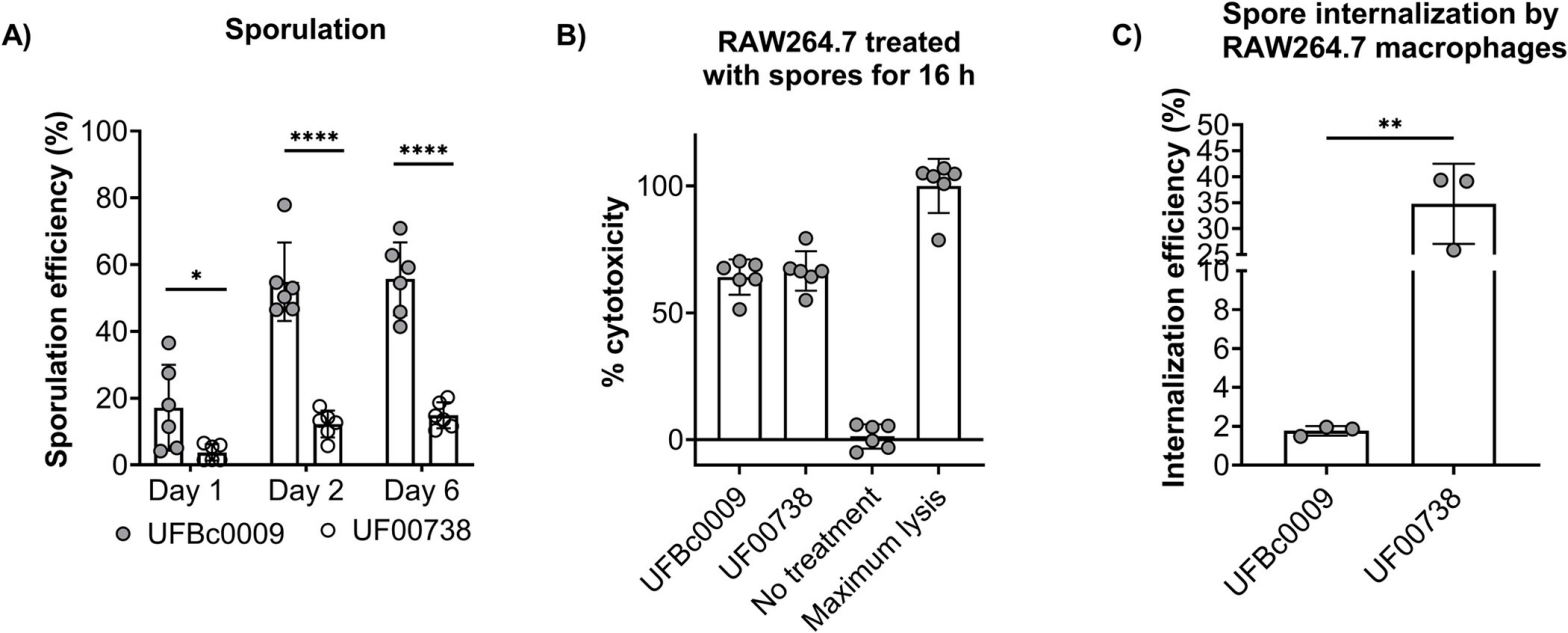
Sporulation, cytotoxicity, and spore internalization of between Bcbva UFBc0009 and *B*. *anthracis* Ames UF00738 in RAW264.7 cells. (A) Number of spores from Bcbva UFBc0009 and *B*. *anthracis* Ames UF00738 produced in HIB broth at 1-, 2-, and 6 day post incubation. At each time point, the number of total spores from Bcbva UFBc0009 (filled circles) and *B*. *anthracis* Ames UF00738 (hollow circle) strains were enumerated, and the sporulation efficiency was calculated as the percentage of spores relative to the total number of bacteria. The data are presented as the mean ± SEM (standard error of the mean). The graphs were the average of 2 independent experiments carried out in triplicate. (B) Cytotoxicity measured in RAW264.7 macrophages infected with Bcbva UFBc0009 and *B*. *anthracis* Ames UF00738 spores at 16-hour post infection. No treatment samples were RAW264.7 cells incubated in media only and served as a vehicle control, while maximum lysis samples were RAW264.7 cells treated with 10X lysis buffer and served as a maximum LDH release control. Six replicates for each strain were tested. The data were presented as the mean ± SEM. (C) The internalization efficiency of RAW264.7 macrophages infected with Bcbva UFBc0009 and *B*. *anthracis* Ames UF00738 spores. Three replicates per strain were examined. The recovered bacteria were identified and calculated as the relative percentage of the initial inoculum. The data were presented as the mean ± SEM. The significance between Bcbva UFBc0009 and *B*. *anthracis* Ames UF00738 strains were determined by unpaired t-test with * = *ρ*<0.05, ** = *ρ*<0.01, **** = *ρ*<0.0001.

Spores of Bcbva and *B*. *anthracis* are considered the infectious particles of anthrax. The sporulation efficiency of Bcbva UFBc0009 and *B*. *anthracis* Ames UF00738 spores was calculated as the percentage of spores relative to the total number of bacteria on day 1, day 2, and day 6 (Fig 1A). Bcbva UFBc0009 sporulated significantly more than *B*. *anthracis* Ames UF00738 from day 1 to day 6. The relative percentage of both Bcbva UFBc0009 and *B*. *anthracis* Ames UF00738 spores gradually increased from day 1 to day 6. Comparing these two strains, Bcbva UFBc0009 sporulated more rapidly than *B*. *anthracis* Ames UF00738. The LDH mediated cytotoxicity assay revealed the cytolytic activities of RAW264.7 macrophages treated with Bcbva UFBc0009 and *B*. *anthracis* Ames UF00738 were 64 and 66%, respectively. The study indicated there was no difference in the level of cytotoxicity between groups (Fig 1B). Following phagocytosis, spores germinated and replicated inside of macrophages, leading to cell death due to the robust secretion of anthrax toxins. This suggests the two strains share similar levels of virulence relative to the toxins linked to cell death released by vegetative cells. Next, RAW264.7 macrophages were infected with Bcbva UFBc0009 and *B*. *anthracis* Ames UF00738 spores at an MOI of 20 to identify differences in internalization efficiencies. At 2 hours post-infection, both Bcbva UFBc0009 and *B*. *anthracis* Ames UF00738 were internalized by RAW264.7 macrophages, however, the internalization efficiency of Bcbva UFBc0009 spores was significantly lower than *B*. *anthracis* Ames UF00738 spores (1.75% for Bcbva UFBc0009 vs 34.80% for *B*. *anthracis* Ames UF00738) (Fig 1C).

### Secretion of protective antigen (PA) by Bcbva compared to *B*. *anthracis* Ames

**Fig 2:**
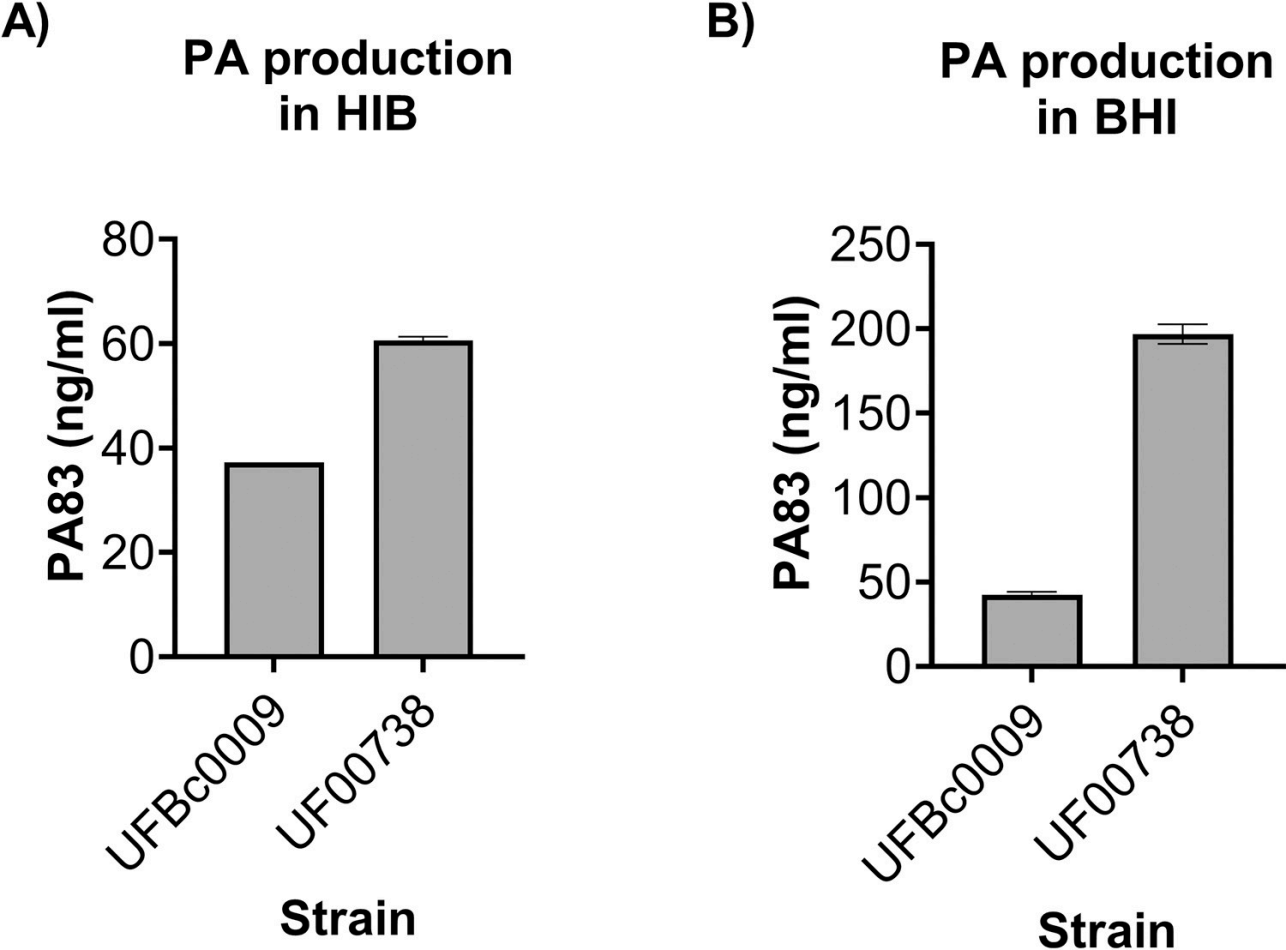
Secretion of PA by Bcbva UFBc0009 and *B*. *anthracis* Ames UF00738. The average amount of PA secreted by either Bcbva UFBc0009 or *B*. *anthracis* Ames UF00738 in HIB (A) and BHI (B) broths at 24 h post-incubation. Two replicates per strain were tested. The data were presented as the mean ± SEM.

Secretion of PA by Bcbva UFBc0009 and *B*. *anthracis* Ames UF00738 in stationary phase HIB and BHI cultured supernatants was measured by ELISA. The amount of PA83, the 83-kDa form of PA, produced by Bcbva UFBc0009 and *B*. *anthracis* Ames UF00738 in HIB broth after 24 h growth was 37.26 and 60.58 ng/ml, respectively (Fig 2A). The average amount of PA secreted by Bcbva UFBc0009 and *B*. *anthracis* Ames UF00738 in BHI broth after 24 h growth was 42.48 and 196.78 ng/ml (Fig 2B). During the stationary phase, the bacterial population size is stable, and the growth rate slows down or even stops owing to nutrient limitation and buildup of waste products, decreasing metabolic activities and synthesis of cellular components. For statistical analysis, we assumed that these data were not normally distributed due to a small sample size (n = 2). The nonparametric test was used to analyze the differences of PA productions between two groups. The results showed that *B*. *anthracis* Ames UF00738 tended to secrete more PA83 than Bcbva UFBc0009 in both culture media, but not at a significant difference (*ρ*<0.333).

### Quantification of attached and secreted PDGA capsule produced by Bcbva

**Fig 3:**
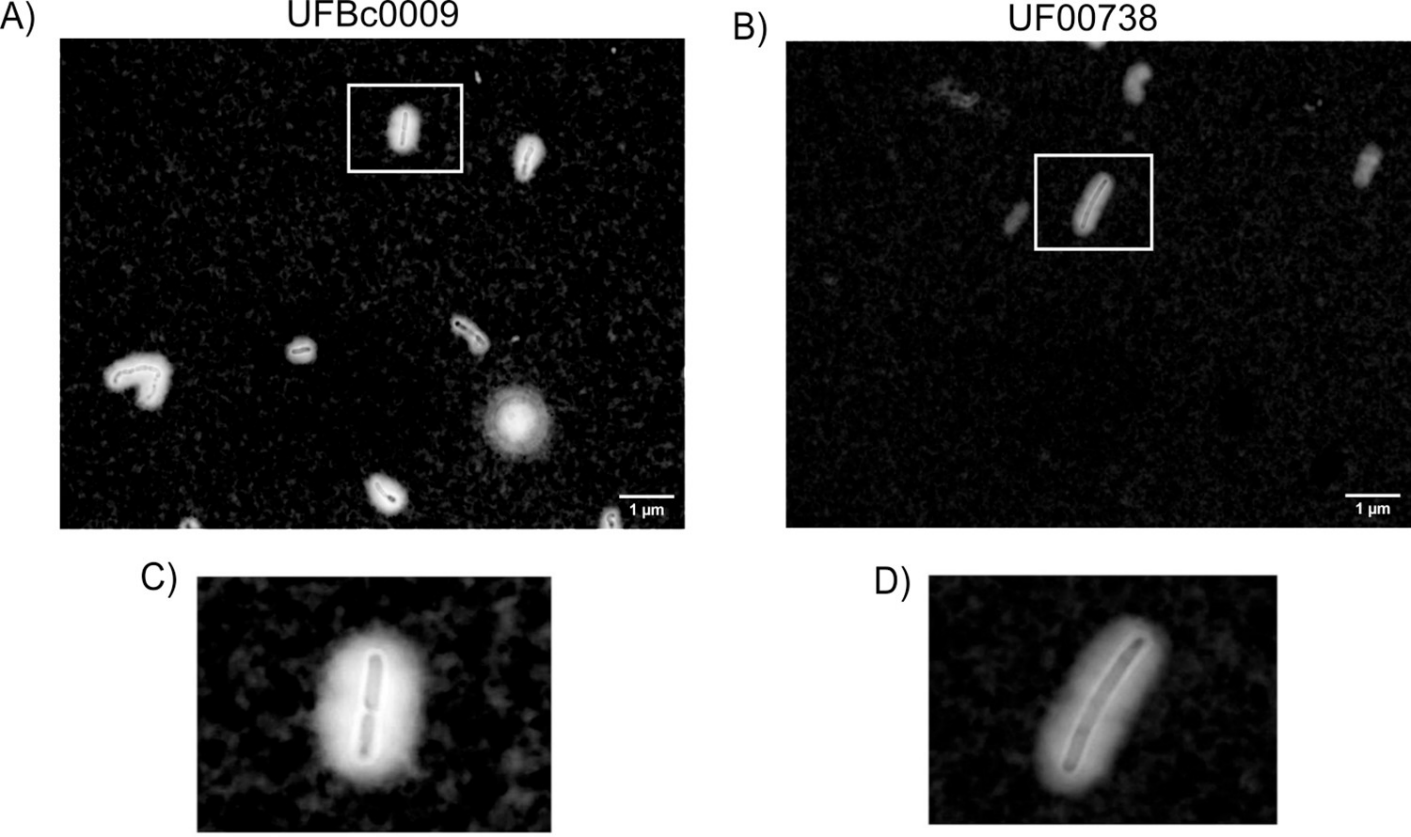
Comparing capsule production between Bcbva UFBc0009 and *B*. *anthracis* Ames UF000738. Bacteria were grown on NBY plates supplemented with 0.7% (v/v) sodium bicarbonate and 20% CO_2_ at 37°C overnight. Bcbva UFBc0009 (A) and *B*. *anthracis* Ames (B) vegetative cells were stained with India ink and examined under 100x oil imersion magnification. Insets magnify Bcbva UFBc0009 (C) and *B*. *anthracis* Ames (D) vegetative cells surrounded by capsules.

**Fig 4:**
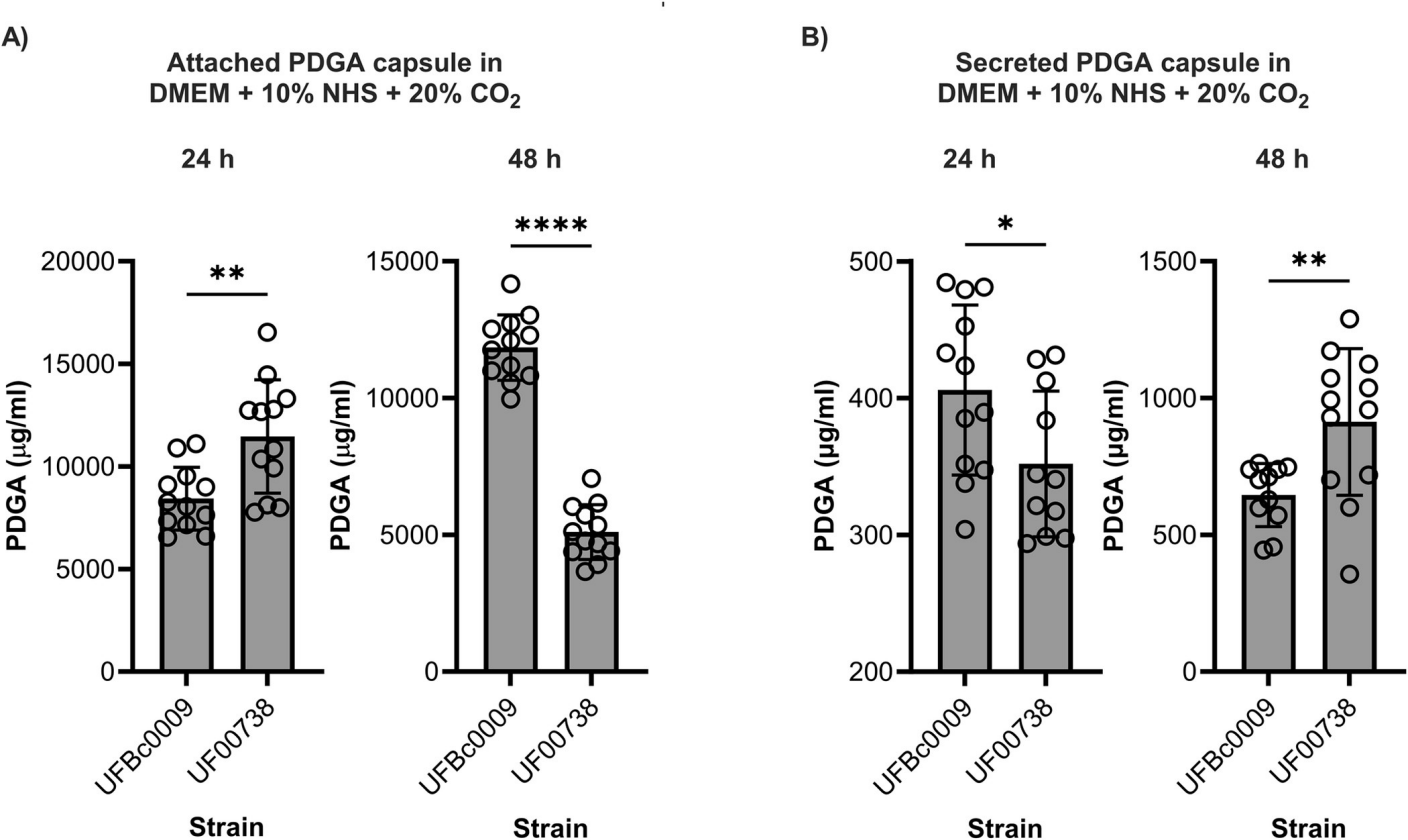
Attached and secreted PDGA capsule produced by Bcbva UFBc0009 compared to *B*. *anthracis* Ames UF00738. The average amount of attached (A) and secreted (B) PDGA capsule produced either by Bcbva UFBc0009 or *B*. *anthracis* Ames in DMEM+10% (v/v) NHS and 20% CO_2_ at 24 and 48 h post incubation. The cultures were grown in triplicate, and two replicates per strain were tested with ELISA. Two-independent experiments were performed, and all data were presented as mean ± SEM. The significance between strains were determined by unpaired t-test with * = *ρ*<0.05, ** = *ρ*<0.01, **** = *ρ*<0.0001.

Bcbva UFBc0009 and *B*. *anthracis* Ames UF00738 were grown at 37°C on bicarbonate agar in a CO_2_-enriched environment for 24 and 48 h to induce capsule formation. Twenty four hours post incubation, the colonies of Bcbva UFBc0009 and *B*. *anthracis* Ames UF00738 appeared mucoid on the agar plates. Capsule produced from vegetative cells of Bcbva UFBc0009 and *B*. *anthracis* Ames UF00738 were visualized by staining with India ink under light microscopy. Both Bcbva UFBc0009 (Fig 3A and 3C) and *B*. *anthracis* Ames UF00738 (Fig 3B and 3D) were encapsulated and showed highly refractile clear zones surrounding bacterial cells against a dark background due to exclusion of India ink by the bacterial capsule. Bcbva UFBc0009 had short-chain rod shaped vegetative cells, while *B*. *anthracis* Ames UF00738 showed long rod-shaped with squared ends arranging in a chain of cells. Qualitatively, Bcbva UFBc0009 produced thicker capsule on the surface of vegetative cells than *B*. *anthracis* Ames UF00738. The amount of attached and secreted PDGA capsule produced by Bcbva UFBc0009 and *B*. *anthracis* Ames UF00738 was quantified by ELISA using anti-capsule IgM. After 24 hours, Bcbva UFBc0009 produced 8,436 μg/ml of attached capsule, while *B*. *anthracis* Ames UF00738 produced a higher amount at 11,460 μg/ml (Fig 4A). At 48-h post incubation, the level of attached capsule from Bcbva UFBc0009 increased to 11,845 μg/ml, whereas *B*. *anthracis* Ames UF00738 decreased to 5,105 μg/ml (Fig 4A). The average amount of attached PDGA capsule produced by *B*. *anthracis* Ames UF00738 was significantly higher than Bcbva UFBc0009 at the 24 h timepoint (*ρ*<0.01). At 48 h, the trend was reversed: Bcbva UFBc0009 had significantly higher amounts of attached PDGA capsule than *B*. *anthracis* Ames UF00738 (*ρ*<0.0001).

The average amount of secreted PDGA capsule produced at 24 h by Bcbva UFBc0009 (405.9 μg/ml) was significantly higher than *B*. *anthracis* Ames UF00738 (351.9 μg/ml) (Fig 4B; *ρ*<0.05). At the 48 h time point, the levels of secreted capsule from both Bcbva UFBc0009 and *B*. *anthracis* Ames UF00738 were higher than the 24 h time point, with *B*. *anthracis* Ames UF00738 secreting more capsule (912.7 μg/ml) than Bcbva UFBc0009 (645.4 μg/ml) (Fig 4B). *B*. *anthracis* Ames UF00738 secreted significantly more capsule than Bcbva UFBc0009 at 48 h post incubation (*ρ*<0.01). Additionally, the average level of secreted capsule from both Bcbva UFBc0009 and *B*. *anthracis* Ames UF00738 was lower than attached capsule.

### Bcbva has a lower lethal dose than *B*. *anthracis* in the *Galleria mellonella* larvae infection model

**Fig 5:**
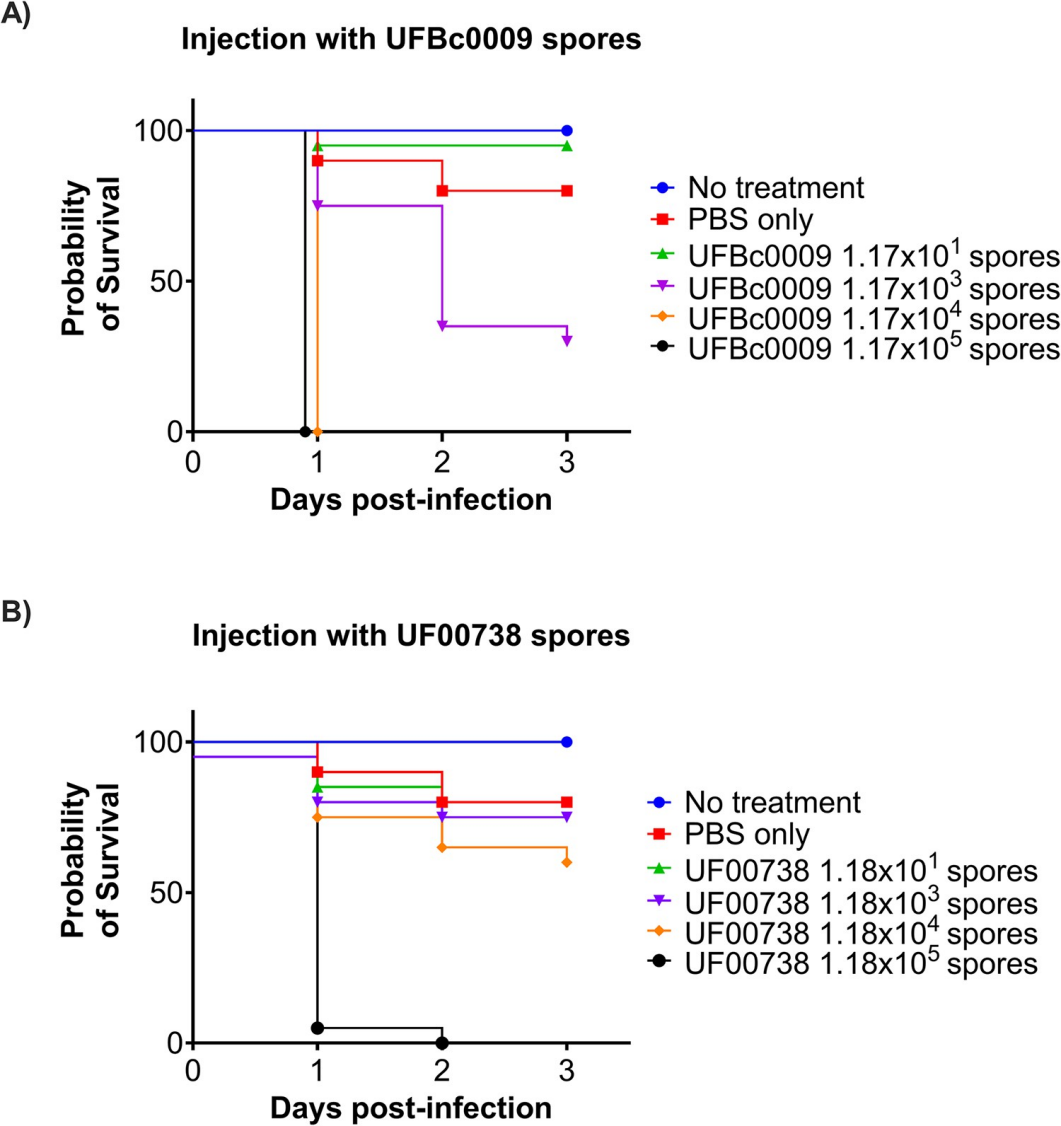
Bcbva UFBc0009 virulence in the *Galleria mellonella* infection model. The survival of wax moth worms was monitored at 24-, 48-, and 72 hour post infection and scored by lack of movements when prodding. Spore dosage and survival time of larvae infected with (A) Bcbva UFBc0009 and (B) *B*. *anthracis* Ames UF00738 spores are presented as Kaplan-Meier survival curves to visualize the probability of survival versus time.

Our studies revealed *G*. *mellonella* larvae did not survive after 24 h when they were infected with 1.17x10^4^ spores of Bcbva UFBc0009, whereas those infected with *B*. *anthracis* Ames UF00738 at 1.18x10^4^ spores survived to 72 hours post infection (Fig 5). Bcbva UFBc0009 and *B*. *anthracis* Ames UF00738 killed larvae in a dose-dependent manner as shown in the LD_50_ curves (Fig 5). The 3-day LD_50_ after hemocoel injection was 9.21x10^2^ spores for *G*. *mellonella* larvae infected with Bcbva UFBc0009 spores (Fig 5A) and 2.24x10^4^ spores for *B*. *anthracis* Ames UF00738 (Fig 5B). The LD_50_ of Bcbva UFBc0009 was 24-fold less than that of *B*. *anthracis* Ames UF00738 in wax moth worms. The nonlinear fit analysis was used to determine the differences between LD_50_ curves, and the result showed that the two LD_50_ values are different as we rejected the null hypothesis that the LD_50_ values were the same for all data set with a *ρ* value of 0.0015. These data suggest that Bcbva UFBc0009 is significantly more virulent than *B*. *anthracis* Ames UF00738 in the *G*. *mellonella* larvae (*ρ*<0.01). Notably, the worms were found to experience some trauma induced by injection: the survival rate from PBS-injected control group was 80% compared to non-injected control group. The survival rate of those groups either infected with 1.17x10^1^ Bcbva UFBc0009 or 1.18x10^1^ *B*. *anthracis* Ames UF00738 spores were found to be like the PBS-injected control group, indicating that the cause of death at these low doses was most likely due to injection injury.

### The LD_50_ of Bcbva in intranasally challenged guinea pigs and efficiency of the Sterne live spore vaccine

**Fig 6:**
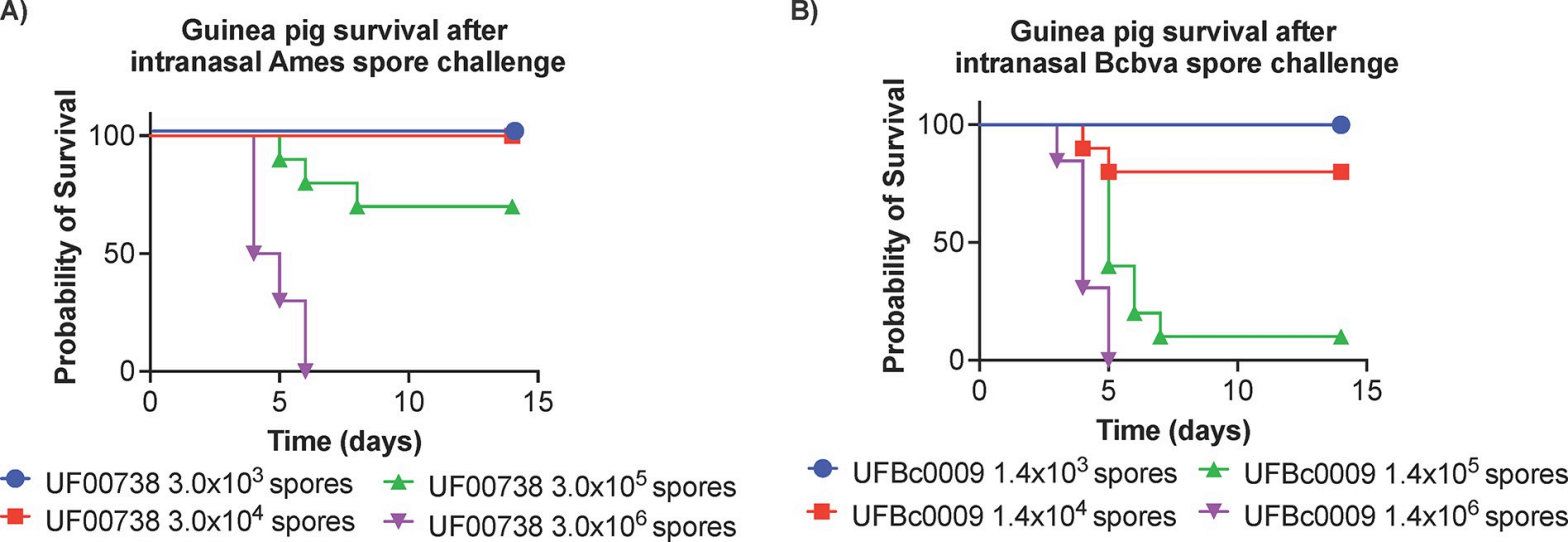
LD_50_ survival of guinea pigs infected with Ames or Bcbva UFBc0009. Kaplan-Meier Survival curves of guinea pigs challenged with the indicated doses of purified Bcbva spores. The number of purified spores were back titrated from the inoculum and used to determine the Ames (A) and Bcbva (B) LD_50_ in guinea pigs. The intranasal 14-day LD_50_ was calculated as 3.61 x10^5^ spores for Ames and 3.49x10^4^ spores for Bcbva UFBc0009.

**Fig 7:**
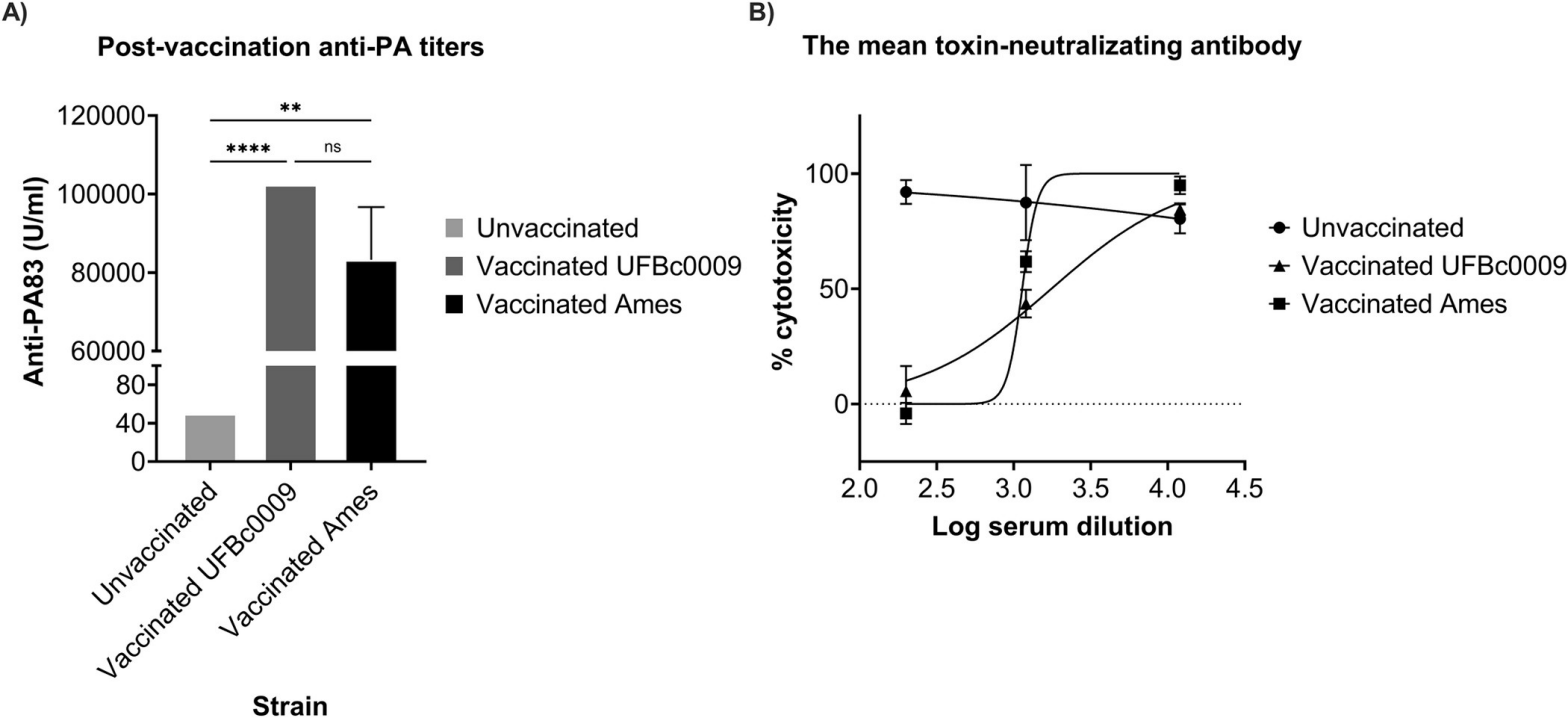
Anti-PA antibody levels and toxin neutralization activities in guinea pigs post vaccination. At 4 week post-vaccination, anti-PA titers (A) and toxin neutralization antibody (B) from vaccinated guinea pigs before being challenged with Bcbva UFBc0009 and *B*. *anthracis* Ames UF00738 spores were quantified. The anti-PA levels were presented as the mean ± SEM. The significance between groups were determined by a Kruskal-Wallis test with Dunn’s multiple comparison test with ** = *ρ*<0.01, **** = *ρ*<0.0001. Toxin neutralizing activities were presented as the mean ± SEM and graphed on a log scale.

**Fig 8:**
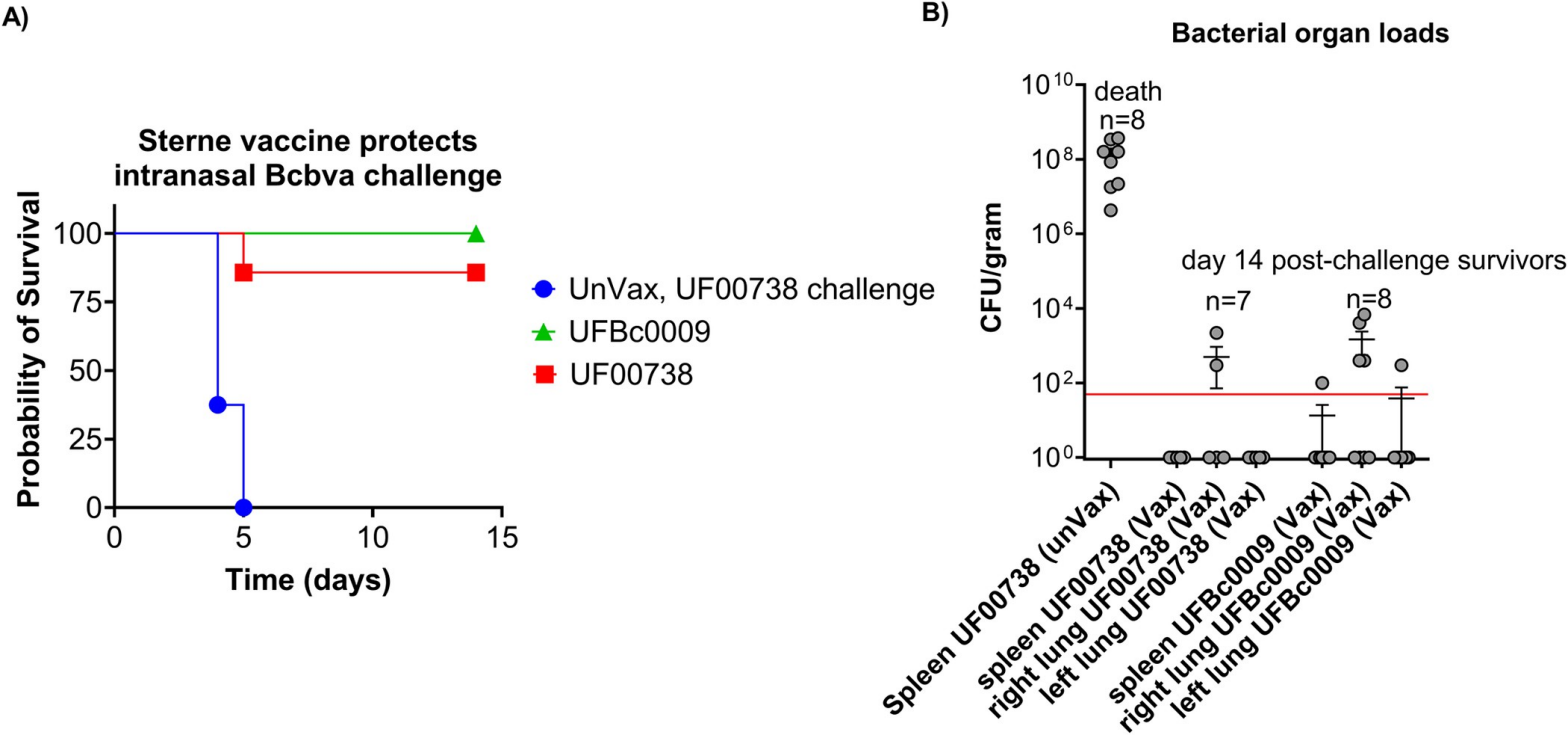
Vaccine efficacy of Sterne against Bcbva UFBc0009 and *B*. *anthracis* Ames UF00738 spores in the intranasal guinea pig model. (A) Survival of vaccinated and unvaccinated guinea pigs following intranasal challenge with 100x LD_50_ spores of indicated strains. Sterne vaccinated guinea pigs infected with Bcbva UFBc0009 spores (green triangle and line) and *B*. *anthracis* Ames UF00738 spores (red square and line) compared to unvaccinated guinea pigs infected with *B*. *anthracis* Ames UF00738 spores (blue dot and line). The log-rank (Mantel–Cox) test was used to determine the significance between vaccinated and unvaccinated groups. (B) The spleens and lungs were collected at 14 days post challenge or at moribundity. Each dot represents data from a single guinea pig with mean ± SD. The limit of detection was shown in the graph (red line).

Median lethal dose of Bcbva UFBc0009 was evaluated in guinea pigs via intranasal route of infection, and the results were compared to *B*. *anthracis* Ames at the Rocky Mountain Regional Biosafety Laboratory at Colorado State University. The LD_50_ of *B*. *anthracis* determined in this study was consistent with a previous report in guinea pigs following inhalation exposures [74]. Guinea pigs became moribund on day 3, and there was no survival when animals were infected at the highest dose of Ames or Bcbva (Fig 6A and 6B, respectively). The intranasal LD_50_ of Bcbva UFBc0009 in guinea pigs was 3.49x10^4^ spores which was 10.3-fold lower than found for *B*. *anthracis* Ames in this study (LD_50_ = 3.61x10^5^ spores, as determined by Probit survival calculations, Fig 6A) and 3.4-fold lower than previously reported for *B*. *anthracis* Ames (LD_50_ = 1.2x10^5^ spores) [74]. Next, the efficacy of the Sterne vaccine against Bcbva UFBc0009 was evaluated in the guinea pig model. At 4 weeks post-vaccination, sera from all vaccinated guinea pigs tested positive for PA-specific IgG as measured by ELISA. The average anti-PA titers from vaccinated animals before being challenged with either Bcbva UFBc0009 or *B*. *anthracis* Ames UF00738 spores were 102,400 and 83,250 ng/ml, respectively (Fig 7A). The antibody levels to PA in the vaccinated animals were significantly higher when compared to unvaccinated controls but were not significantly different between the two challenge groups. Toxin neutralization activity from vaccinated guinea pigs pre-challenged with Bcbva UFBc0009 ranged from 53.3%-61.7% with a mean of 56.4% (cytotoxicity measurement of 39.3% to 47.7% with a mean of 43.6%) while pre-challenged *B*. *anthracis* Ames vaccinated group ranged from 35.0%-42.4% with a mean of 38.2% (cytotoxicity measurement of 58.6% to 65.0% with a mean of 61.8%) at a sera dilution of 1:1,200 (Fig 7B). Pooled serum from unvaccinated guinea pigs diluted 1:1,200 exhibited mean toxin neutralization activity at 13.6% that ranged from 1% to 24.1% (cytotoxicity measurement of 65.9% to 99.0% and mean of 86.4%). The 50% cellular protection (ED_50_) of vaccinated guinea pigs pre-challenged with Bcbva UFBc0009 and *B*. *anthracis* Ames were at 1,756- and 1,136- fold dilutions, respectively. The data indicated that the ability of antibody to neutralize the *in vitro* cytotoxicity of PA and LF in vaccinated animals was higher than unvaccinated controls. At 14 days post challenge, the results showed 100% and 85% of Sterne vaccinated guinea pigs survived after intranasal spore challenge with 100x LD_50_ of either Bcbva UFBc0009 or B. *anthracis* Ames UF00738, respectively (Fig 8A). The survival of unvaccinated animals challenged with B. *anthracis* Ames UF00738 was significantly different from animals vaccinated then challenged with Bcbva UFBc0009 (*ρ*<0.0001) and B. *anthracis* Ames UF00738 (*ρ*<0.001). Only one vaccinated guinea pig was moribund 5 days post-infection with *B*. *anthracis* Ames UF00738. All unvaccinated control guinea pigs were moribund by day 5 post challenge. Sterne vaccinated guinea pigs were completely protected from intranasal Bcbva spore challenge. Spleens of unvaccinated animals contained viable bacteria and ungerminated spores, while infected animals from the vaccinated group demonstrated some levels of organ clearance (Fig 8B).

## Discussion

*Bacillus cereus* biovar *anthracis* is a genetic near neighbor of *B*. *anthracis* and has been a cause of anthrax-like disease in livestock and wildlife across sub-Saharan Africa for decades [24]. Bcbva evolved from *B*. *cereus* by acquiring virulence plasmids almost identical to those found in *B*. *anthracis* [30]. The ability of Bcbva to cause disease is attributed to the production of secreted anthrax toxins and the PDGA capsule [75,76]. Here, we demonstrated Bcbva UFBc0009 sporulated more rapidly and to a higher magnitude compared to *B*. *anthracis* Ames UF00738. This finding is consistent with a recent study that compared Bcbva isolated from chimpanzees in Cameroon and Côte d’Ivoire to *B*. *anthracis* strains [39]. A previous study showed wild, low passage *B*. *anthracis* strains sporulate faster than laboratory *B*. *anthracis* strains which have been repeatedly sub-cultured due to the limitation of nutrients [77]. One reason for this is laboratory strains consume nutrients slower than wild strains, leading to a delay in sporulation time [77]. Therefore, it is possible the wild, low passage Bcbva used in this study can assimilate available nutrients faster than the laboratory *B*. *anthracis* Ames UF00738 strain resulting in rapid sporulation.

Furthermore, our data provide additional understanding of sporulation in Bcbva compared to *B*. *anthracis*. *Bacillus anthracis* spores persist in the locally infectious zone (LIZ) for extended periods of time; recent work suggests approximately 10 years where LIZs are undisturbed and decay naturally [78]. The rapidity of spore formation and quantity of spores may serve to efficiently transmit Bcbva in its natural conditions. Studies also demonstrate Bcbva is distributed and transmitted by necrophagous flies [24,79]. This parallels a report of *B*. *anthracis* spores being mechanically spread from anthrax carcasses by flies [80], such as in Texas and elsewhere where this occurs during outbreaks. Spore contaminated carcasses are highly valuable sources of nutrients for necrophagous fly replication. Following the case multiplier hypothesis, necrophagous flies can spread viable bacteria from a carcass through the surrounding environment forming a wide area LIZ beyond the immediate carcass site, especially in the first days after host death [80,81]. A previous study estimated a single blow fly can distribute *B*. *anthracis* spores from a carcass via fly deposits of up to 8.62 x 10^5^ spores per day [9]. This process leads to increased exposure risk for other animals and contributes to wildlife mortality during an outbreak. Currently, the source of wildlife infection with Bcbva in Africa remains unclear. In Taï National Park, 5% of randomly caught carrion flies were PCR positive for plasmid (*pag* and *capB*) and chromosomal (genomic island IV) Bcbva markers, and many of these flies carried viable Bcbva spores [24]. A recent study showed flies associated with Bcbva-infected mangabey monkeys (*Cercocebus atys atys*) in Taї National Park contained Bcbva DNA, and Bcbva were recovered from the PCR-positive flies [79]. It is conceivable that flies may transport Bcbva as spores or vegetative cells from externally contaminated mouthparts and legs, or from ingesting spores and/or vegetative cells. Further studies are needed to examine the relationship between Bcbva and flies in Taї National Park.

The host-pathogen interaction between macrophages and *B*. *anthracis* is initiated by recognition followed by elimination of the pathogen [82]. During the initial stage of infection, macrophages play an essential role in the pathogenesis of *B*. *anthracis* infection as they are responsible for phagocytosis, while simultaneously functioning as a vehicle to transport *B*. *anthracis* spores to the draining lymph nodes [83]. Macrophages cannot directly kill the *B*. *anthracis* spores but rather kill the vegetative bacilli once spores germinate [84]. Upon pulmonary instillation, alveolar macrophages engulf and transport the spores to tracheobronchial lymph nodes where spores germinate and form vegetative cells within the phagolysosome of phagocytic cells [85]. Some vegetative cells are killed, while others remain viable and escape from phagolysosome into the cytoplasm [85]. The bacilli multiply rapidly and secrete toxins resulting in death of macrophages. When the bacillary replication exceeds the capacity of regional lymph nodes, the bacteria enter the bloodstream where they grow extracellularly to levels leading to systemic infection [86]. In advanced anthrax infection, the number of vegetative cells is as high as 10^8^ bacteria/ml of blood [5] suggesting the innate immune responses is overwhelmed. Our data demonstrated that the cytolytic activities of RAW264.7 macrophages infected with Bcbva UFBc0009 and *B*. *anthracis* Ames UF00738 spores at 16 hours post infection were comparable. This pattern coincided with the secreted PA levels in both HIB and BHI broths at 24 h post inoculation. The amount of PA generated by Bcbva UFBc0009 and *B*. *anthracis* Ames UF00738 did not differ substantially between medium types. Cytotoxicity levels indicate toxin release, as *B*. *anthracis* vegetative cells produce toxins and proteases during replication, resulting in tissue lysis further facilitating the dissemination of bacteria to deeper tissue layers [48,58]. The specific mechanisms by which Bcbva and *B*. *anthracis* spores survive, and multiply within macrophages are still unclear. The kinetics of bacterial multiplication inside macrophages have yet to be determined. To compare the efficiency of spore uptake by macrophages, we demonstrated Bcbva spores could be internalized and survive within mouse macrophages at the initial stage of infection like *B*. *anthracis*. Nevertheless, *B*. *anthracis* Ames spores had significantly higher internalization efficiency than Bcbva spores. The low level of recovered bacteria in macrophages infected with Bcbva UFBc0009 is possibly due to differences in components and functions of the exosporium, the hairy-like outermost layer of the spores. In addition, the exosporium of *B*. *anthracis* appears to play an essential role in protecting the spores from macrophage-mediated killing [84]. The exosporium regulates intracellular survival and influences spore germination in *B*. *anthracis* infected macrophages [82]. Upon phagocytosis and spore germination, the kinetics of capsule production inside phagocytic cells is still not clear. The differences in the encapsulation of Bcbva and *B*. *anthracis* could affect the intracellular survival inside macrophages [87]. The additional production of hyaluronic acid to PDGA capsules in Bcbva may give the bacteria a survival advantage by inhibiting phagocytosis and enabling evasion from the innate immune system. Like *B*. *anthracis*, the initial interaction of Bcbva spores with the infected macrophages may play role in survival of Bcbva spores from the macrophage killing mechanisms. However, the ability of the exosporium and capsules to promote intracellular survival of Bcbva spores within infected macrophages has yet to be elucidated fully. Additionally, it is important to note the limitations of cytotoxicity and internalization assays in this study. The experiments were designed based on previously published literature [48–51,53]. Further microscopy and molecular studies to directly assess spore germination and replication kinetics inside macrophages are necessary to move past the limits of cytotoxicity and internalization assays seen here. The current study demonstrated that the production of PA by Bcbva UFBc0009 at the stationary phase appeared to be lower than *B*. *anthracis* Ames, but not significant differences. This finding is consistent with the study comparing *B*. *anthracis* and Bcbva isolates grown in bicarbonate/CO_2_ conditions, in which the levels of PA secreted by Bcbva were somewhat lower than that of *B*. *anthracis* [31]. PA is an important virulence factor of *B*. *anthracis* and acts as the major protective immunogen during vaccination [88]. One feature of anthrax vaccination is the production of PA can induce protective immunity against both lethal and edema toxins. In fact, PA is the major immunogenic constituent of the current available human vaccines for anthrax [14,89]. A previous study showed mice vaccinated with formaldehyde-inactivated *B*. *anthracis* spores and recombinant PA vaccine were protected against Bcbva spores via subcutaneous inoculation [31]. As a PA-producing bacterium, it is feasible that PA secreted by Bcbva can be used as a target for new therapeutic approaches against anthrax in livestock and wildlife in Africa.

When grown on NBY medium supplemented with bicarbonate in 20% CO_2_ at 37°C, Bcbva UFBc0009 vegetative cells synthesized capsule on the cell surface like *B*. *anthracis* Ames UF00738 vegetative cells. Bcbva UFBc0009 expressed slightly higher amounts of capsule than *B*. *anthracis* Ames UF00738 when observed under the light microscope. These findings are consistent with a previous study that found Bcbva expresses both hyaluronic acid and PDGA capsules [31]. By measuring the level of PDGA capsule production, we showed Bcbva UFBc0009 produced significantly more attached PDGA capsule than *B*. *anthracis* Ames UF00738 once bacterial cultures were grown in DMEM supplemented with 10% (v/v) NHS and 20% CO_2_. PDGA capsule is a poor immunogen, is resistant to degradation, and has comparable properties to T cell-independent polysaccharides [86] while hyaluronic acid is a poor immunogen due to antigen mimicry of the mammalian extracellular matrix. During infection, PDGA capsule produced by bacteria inhibits complement deposition and opsonization by macrophages [90,91]. Purified capsule can inhibit the bactericidal activity of alpha defensin in neutrophils *in vitro* [92]. Taken together, it is possible Bcbva can survive better than *B*. *anthracis* in phagocytic cells. However, capsule dependent virulence is unclear, as a study showed that a capsule negative Bcbva cured of pBCXO2, while retaining pBCXO1 and still capable of producing hyaluronic acid capsule, remained highly virulent in mice challenged intranasally when compared to an acapsular, virulent *B*. *anthracis* Sterne (pXO2 negative) strain [31]. Previous literature suggested hyaluronic acid capsule encoded by the pBCXO1 plasmid may contribute to a more successful infection by providing higher chance for survival of bacterium in mammalian hosts thus favoring the dispersal of pathogens [31,93]. For *B*. *cereus* G9241, a pathogenic strain causing anthrax-like disease, the presence of hyaluronic acid capsule may facilitate *in vivo* infection [93].

Challenge and LD_50_ experiments were conducted in animal models to characterize the virulence of the earliest known Bcbva isolates. A survival assay was performed to compare the virulence between Bcbva UFBc0009 and *B*. *anthracis* Ames UF00738 in *G*. *mellonella* larvae and guinea pigs. We demonstrated that *G*. *mellonella* results correlate to virulence levels of Bcbva and *B*. *anthracis* Ames in small mammal models. We discovered Bcbva UFBc0009 from Taї National Park is significantly more virulent than *B*. *anthracis* Ames UF00738. The 3-day LD_50_ of Bcbva UFBc0009 was 24-fold lower than *B*. *anthracis* Ames UF00738 in the *G*. *mellonella* model. Akin to *G*. *mellonella* infection, intranasal LD_50_ of Bcbva UFBc0009 in guinea pigs was 3.4-fold lower than reported for *B*. *anthracis* Ames [74]. Our findings in guinea pigs suggest Bcbva from Côte d’Ivoire has a lower intranasal LD_50_ than a neighboring strain in Cameroon [31], however, both had lower LD_50_ than the *B*. *anthracis* strains used for comparison.

Currently, the Sterne vaccine is the most common vaccine used in wildlife and livestock to prevent anthrax [94–98]. Its efficacy in guinea pigs was examined in this study, as they were vaccinated with the Sterne vaccine and later intranasally challenged with spores from Bcbva UFBc0009. The antibody profiles in these vaccinated animals revealed all pre-challenge vaccinated guinea pigs had significantly higher anti-PA levels compared to non-vaccinated animals. The protection was then tested *in vitro* by the sera’s ability to neutralize the cytotoxic effect of anthrax lethal toxin against RAW264.7 cells. The results showed neutralizing antibody titers correlated with anti-PA titers by ELISA. The increase in anti-PA titers and the lethal toxin neutralization antibodies in vaccinated guinea pigs conferred notable protective immunity to anthrax. A previous PA vaccine study showed protection against Bcbva demonstrated all mice immunized with recombinant PA and formaldehyde-inactivated *B*. *anthracis* spores of a genetically detoxified Sterne strain (RPLC 2) survived subcutaneous infection with Bcbva or *B*. *anthracis* spores [31]. In the current study, the efficacy of the Sterne vaccine in protecting against Bcbva was complete and comparable to the challenge with *B*. *anthracis* Ames UF00738 in the guinea pig model. The vaccine protected against Bcbva UFBc0009 and *B*. *anthracis* Ames UF00738 spores, resulting in 8/8 and 7/8 animals surviving, respectively. The Bcbva UFBc0009 group showed a significant increase in survival time of 14 days versus 5 days for the unvaccinated control. However, it should be noted Sterne vaccine did not achieve sterilizing immunity as residual bacteria and ungerminated spores remained in the lungs and spleens at the end of the 14-day study. Several animal models including guinea pigs have shown that a subpopulation of spores may remain ungerminated within the site of infection [72,99–101]. The high level of recovered bacteria in animals challenged with Bcbva UFBc0009 was possibly due to the expression of hyaluronic acid and PDGA capsules which may provide a survival advantage to the bacteria by impeding complement fixation, allowing evasion of host immunity compared to *B*. *anthracis* Ames UF00738. The impacts of host immune responses to spore challenge remain to be elucidated. Vaccination is the standard control of anthrax and can break the cycle of transmission [102]. Several studies have demonstrated that vaccine implementation in exposed susceptible animals and proper discarding of infected carcasses impedes the spread of infection and death, especially in endangered species [95,98]. The only licensed animal vaccine available in the US is the Sterne 34F2 live spore vaccine which can be used in livestock including cattle, sheep, goats, pigs, and horses or off-label use in other species [16]; internationally other Sterne-like strains are often used in vaccine production [103]. Further efficacy studies and risk assessments for the Sterne vaccine are required for those additional species, in particular non-human primates of conservation concern.

When comparing the wild Bcbva UFBc0009 isolate and *B*. *anthracis* Ames UF00738 strain, we found differences in sporulation efficiencies and PDGA capsule production, specifically Bcbva UFBc0009 sporulated and produced more PDGA capsule than *B*. *anthracis* Ames UF00738. *B*. *anthracis* Ames UF00738 spores were internalized by mouse macrophages at higher efficiency than Bcbva UFBc0009 spores. However, the cytolytic activity of infected RAW264.7 macrophages and secreted PA levels in minimal media were comparable between the two strains. Although our data demonstrated significant differences between the two strains, these differences could be strain-specific rather than species-specific. More research is required to understand the role of Bcbva strain diversity in pathogenesis.

Besides host-pathogen interaction, future studies should focus on Bcbva lifecycle outside of host cells. These include the kinetics of replication and sporulation of Bcbva on environmental substrates in the LIZ areas may be important as carrion flies can transport viable Bcbva spores and may amplify cases during an outbreak in Africa. The species most affected by Bcbva appear to be non-human primates living in humid tropical forest environments, whereas *B*. *anthracis* infection is commonly found in ungulates in savanna habitats. Further investigation into LIZ contamination and transmission pathways may provide new preventative methods to control the recurrence of enzootic outbreaks caused by Bcbva in wildlife and livestock. To prevent the future occurrence of anthrax outbreak caused by Bcbva in West and Central Africa, trials of Sterne vaccine in affected livestock, hoofed wildlife, and several non-human primates should be performed.

## Supporting information

S1 Data The excel workbook contains the raw data used to produce the images in each figure of the paper. The tabs in the workbook are labeled Figs 1–2 and 4–8 and contain the data points for each of the indicated figures. Fig 3 is a microscopy image figure so there are no data points for this figure.
(XLSX)
